# 

**Published:** 1858-08

**Authors:** 


### RECEIPTS FOR JOURNAL UP TO SEPTEMBER 16th, 1858.

### Illinois.

| | | | |
|---|---|---|---|
| Dr. Max Myers, | vol. 1, C. J., 1858, | | $2 |
| " A. W. Butler, | " | " | 2 |
| " A. J. Mead, | " | " | 2 |
| " N. Holton, | " | " | 2 |
| " U. G. Stevens, | " | " | 2 |
| " A. A. Barrows, | " | " | 2 |
| " P. W. Hill, | " | " | 2 |
| " Graham & Manly, | " | " | 2 |
| " Ross & Graham, | " | " | 2 |
| " David Prince, | " | " | 2 |
| " B. Wilson, | " | " | 2 |
| " Thos. Winston, | " | " | 2 |
| " John Bowman, | " | " | 1 |
| " J. C. Lowrie, | " | " | 2 |
| " G. W. Phillips, | " | " | 2 |
| " E. Friend, | " | " | 2 |
| " E. E. Webster, | " | " | 2 |
| " P S. Secor, | " | " | 2 |
| " J. R. Hayes, | vol. 6, 1857, | | 4 |
| " F. J. Dickinson, | vol. 6 & 1 C. J. 1857-8, | | 2 |
| " L. Smith, | " | " | 4 |
| " Chester Hard, | " | " | 4 |
| " E. Woodruff, | vol. 5, 6 & 1 C. J. 1856-7-8, | | 6 |

### Michigan.

| | | | |
|---|---|---|---|
| Dr. Alex. D. E Armond, | vol. 1 C. J. 1858, | | $2 |
| " L. W. Lovell, | " | " | 2 |
| " J. F. Williams, | vols. 5, 6 & 1 C. J. 1856-7-8, | | 8 |

### Indiana.

| | | | |
|---|---|---|---|
| Dr. N. H. Canaday, | vol. 1 C. J. 1858, | | $1 |
| " Jeff. Miller, | " | " | 2 |
| " J. H. Newland, | " | " | 2 |
| " Coe & Constant, | " | " | 2 |
| " D. Rea, | " | " | 2 |
| " W. Townsend, | " | " | 2 |
| " W. Vannuys, | " | " | 2 |
| " G. W. Carpenter, | " | " | 2 |

### Minnesota.

| | | |
|---|---|---|
| Dr. C. L. Anderson, | vol. 6, 1857, | $2 |
| " D. W. Hand, | vol. 1 C. J. 1858, | 2 |

### Wisconsin.

| | | | |
|---|---|---|---|
| Dr. A. P. Adams, | vol. 1 C. J. 1858, | | $2 |
| " D. L. Downs, | " | " | 2 |
| " J. Wiley, | " | " | 2 |
| " Rowley Morris, | " | " | 2 |
| " J. B. Moffat, | " | " | 2 |
| " A. Bodenstal, | " | " | 2 |

### Iowa.

| | | | |
|---|---|---|---|
| Dr. H. Love, | vol. 1 C. J. 1858, | | $2 |
| " S. L. Hazen, | " | " | 2 |
| " John Low, | " | " | 1 |

### California.

| | | |
|---|---|---|
| Dr. M. R. Fowler, | vol. 1 C. J. 1858, | $2 |

